# Transmission of COVID-19 on an Inpatient Hospital Prison Unit

**DOI:** 10.1017/ash.2021.87

**Published:** 2021-07-29

**Authors:** Kelsey Witherspoon, Manisha Shah, Justin Smyer, Nora Colburn, Christina Liscynesky, Courtney Hebert, Shandra Day

## Abstract

**Background:** Prison populations have been disproportionally affected by COVID-19, partly due to challenges related to social distancing. Data on viral transmission dynamics in inpatient prison units remain limited. The Ohio State University Wexner Medical Center (OSUWMC) has a 24-bed inpatient prison unit in collaboration with the Ohio Department of Rehabilitations and Corrections (ODRC). The unit has 5 shared rooms holding 4 patients each and 4 single-patient rooms. Several cases of inpatient transmission of COVID-19 were identified on the inpatient prison unit. **Methods:** An IRB-approved retrospective chart review was conducted to evaluate inpatient transmission dynamics of hospital-acquired (HCA) COVID-19. All ODRC patients admitted from March 1 to April 24, 2020, were included. Patients assigned to the prison unit during their hospital stay were evaluated for potential HCA COVID-19, defined as a positive SARS-CoV-2 test ≥4 days after admission. Patient characteristics, testing data, symptoms, aerosol-generating procedures (AGPs), and room assignments were reviewed. Healthcare workers (HCWs) and correction officers (COs) working on the unit who tested positive during this period were identified. **Results:** In total, 142 ODRC patients were admitted during the study period and 89% had a positive SARS-CoV-2 testing prior to or during admission. Also, 61 patients (43%) were assigned to the prison unit. Moreover, 8 patients on the unit met potential HCA COVID-19 definition with 7 linked to 3 distinct clusters. Also, 7 COs had COVID-19 (outside hospital exposure) and 5 HCWs acquired COVID-19 from patient exposure on the unit. In cluster 1, 4 patients admitted to the same room developed HCA COVID-19. A symptomatic index patient not tested on admission given an atypical presentation required CPAP and frequent nebulizer treatments. In cluster 2, 1 patient from cluster 1 was transferred to another room. The new roommate subsequently developed HCA COVID-19. In cluster 3, a symptomatic correctional officer was assigned to 2 patients in a shared room; the patients later developed HCA COVID-19. **Conclusions:** Three patient clusters of HCA COVID-19 on a prison unit were identified. Aerosol transmission potentially played a role in cluster 1. Inpatient transmission within the unit prompted updated guidance for ODRC admissions, including universal SARS-CoV-2 admission testing, excluding patients requiring AGPs from shared rooms, and preemptive isolation for patients from an ODRC facility experiencing a COVID-19 surge. Universal testing was quickly expanded to all inpatient admissions. HCWs and COs were also linked to inpatient transmission, highlighting the importance of strict infection control practices for patient populations who cannot socially distance.

**Funding:** No

**Disclosures:** None

